# Sonographic evaluation of pediatric localized scleroderma: preliminary disease assessment measures

**DOI:** 10.1186/1546-0096-8-14

**Published:** 2010-04-27

**Authors:** Suzanne C Li, Melissa S Liebling, Faridali G Ramji, Sven Opitz, Arun Mohanta, Tatiana Kornyat, Shuzhen Zhang, Molly Dempsey-Robertson, Carsten Hamer, Stephanie Edgerton, Jose Jarrin, Mike Malone, Andrea S Doria

**Affiliations:** 1 Department of Pediatrics, Joseph M Sanzari Children's Hospital, Hackensack University Medical Center, Hackensack, USA; 2 Department of Radiology, Hackensack University Medical Center, Hackensack, USA; 3 Department of Radiology, Children's Hospital of Oklahoma, Oklahoma University Medical Center, Oklahoma City, USA; 4 Department of Radiology, Klinikum Eilbek - Schön Kliniken, Hamburg, Germany; 5 Department of Diagnostic Imaging, Toronto Hospital for Sick Children, Toronto, Canada; 6 Department of Radiology, Texas Scottish Rite Hospital, Dallas, USA

## Abstract

**Background:**

Our earlier work in the ultrasonograpy of localized scleroderma (LS) suggests that altered levels of echogenicity and vascularity can be associated with disease activity. Utrasound is clinically benign and readily available, but can be limited by operator dependence. We present our efforts to standardize image acquisition and interpretation of pediatric LS to better evaluate the correlation between specific sonographic findings and disease activity.

**Methods:**

Several meetings have been held among our multi-center group (LOCUS) to work towards standardizing sonographic technique and image interpretation. Demonstration and experience in image acquisition were conducted at workshop meetings. Following meetings in 2007, an ultrasound measure was developed to standardize evaluation of differences in echogenicity and vascularity. Based upon our initial observations, we have labeled this an ultrasound disease activity measure. This preliminary measure was subsequently evaluated on over 180 scans of pediatric LS lesions. This review suggested that scoring levels should be expanded to better capture the range of observed differences. The revised levels and their definitions were formulated at a February 2009 workshop meeting. We have also developed assessments for scoring changes in tissue thickness and lesion size to better determine if these parameters aid evaluation of disease state.

**Results:**

We have standardized our protocol for acquiring ultrasound images of pediatric LS lesions. A wide range of sonographic differences has been seen in the dermis, hypodermis, and deep tissue layers of active lesions. Preliminary ultrasound assessments have been generated. The disease activity measure scores for altered levels of echogenicity and vascularity in the lesion, and other assessments score for differences in lesion tissue layer thickness and changes in lesion size.

**Conclusions:**

We describe the range of sonographic differences found in pediatric LS, and present our efforts to standardize ultrasound acquisition and image interpretation for this disease. We present ultrasound measures that may aid evaluation of disease state. These assessments should be considered a work in progress, whose purpose is to facilitate further study in this area. More studies are needed to assess their validity and reliability.

## Background

Localized scleroderma (LS) is the most common form of scleroderma in the pediatric age range. For both systemic sclerosis and LS, there is an initial inflammatory phase followed by the replacement of normal tissue structures by abnormal collagen [1]. Although scleroderma refers to hardening of the skin, pediatric LS often affects deeper tissue, including muscle, bone, and some internal organs [2]. Because active disease can persist for years, growing children are at risk for major morbidity including joint contractures, limb length discrepancy, and facial atrophy [3,4].

There are no available laboratory markers or validated measures for monitoring disease activity in localized scleroderma [4]. Disease activity is usually defined as the level or severity of reversible disease manifestations, while damage is defined as irreversible, accumulated changes from prior active disease [5]. Monitoring disease activity can improve patient management, as increased activity may signal a need for increased or intensified treatment, while a sustained decrease may indicate that treatment can be reduced.

Among the different imaging modalities available to evaluate LS, ultrasound shows great promise for aiding clinical assessment of LS disease activity. Several groups have reported that changes in dermal thickness and level of echogenicity correlate with disease activity [6-8]. We have found these alterations also occur in deeper tissue layers such as hypodermis and muscle. Moreover, we have found a range of vascularity differences in the lesion. In some patients, clinical improvement was associated with loss of hyperemia, and worsening with increased hyperemia and hyperechogenicity [9,10].

Studies by other groups of LS and other diseases, where concurrent histological and ultrasound studies were performed, have shown that hypoechogenicity is associated with tissue edema, while hyperechogenicity is associated with infiltration of normal tissue by inflammatory cells, fat cells, or connective tissue [11-14]. Histological studies have found tortuous and enlarged vessels, disturbed vascular architecture, and neovascularization in localized scleroderma lesions [15,16]. Older lesions can show a reduced number of blood vessels because of replacement of normal structures by fibrosis [1]. Increased blood flow has also been detected in active lesions by laser Doppler flowmetry, and suggested by the increased temperature detected in active lesions by thermography [17,18].

Although many studies in several rheumatic diseases have shown ultrasound to be more sensitive than clinical evaluation, its use has been limited because both image acquisition and interpretation are operator dependent. There are no standards for most assessed features making image interpretation potentially subject to great variability between readers. To aid standardization of image interpretation, scoring measures have been generated for sonographic evaluation of different diseases. In rheumatoid arthritis, groups have proposed grading the observed echogenicity features of effusions and synovitis on a 0 to 3 semiquantitative scale (0 is no abnormality, 1 mild or minimal, 2 moderate, and 3 marked or extensive)[19,20]. Similarly, semiquantitative scoring of power Doppler signal from imaged joints has been proposed [19], with good agreement found between semiquantitative (0 to 3 scale) and quantitative scoring (based upon pixel count) in one study [21].

In other diseases, groups have proposed grading the level of echogenicity. For evaluating patients with neuromuscular, primary myopathy, and/or inflammatory muscle disease, Heckmatt et al proposed scoring muscle echogenicity on a 1 to 4 semiquantitative scale; good correlation was found between the level of muscle echogenicity and the severity of the muscle biopsy pathology [22]. Scoring the level of echogenicity of the parotid gland and breast tissue masses has also been suggested [23,24].

As echogenicity level, vascularity, and tissue thickness vary across anatomic sites and between individuals [14], it is important to select appropriate comparison sites or standards when determining if the observed signals are abnormal. For Sjogren's syndrome, parotid echogenicity was compared to thyroid echogenicity, and for muscle diseases, muscle echogenicity was compared to bone [22,23]. Because LS, by definition, affects a limited region of the body, this disease may be particularly suitable for evaluation by ultrasound as each patient can serve as his or her own control. We evaluate the LS lesion in comparison to an uninvolved focus, ideally the normal contralateral site, to enable accurate assessment of changes in these parameters.

We have formed a multi-center, multi-disciplinary group, LOCUS (Localized scleroderma Clinical and Ultrasound Study group) that has been working to develop disease assessment tools for LS. The member sites are predominantly based in CARRA (Childhood Arthritis and Rheumatology Research Alliance), a North American pediatric rheumatology research alliance. To facilitate standardization of image acquisition for localized scleroderma, we have held workshop meetings to allow review and standardization of technique. To standardize image interpretation, we have generated scoring measures for evaluating differences in echogenicity, vascularity, and tissue thickness based upon our review of over 180 lesion scans from 21 patients. These measures should be considered a work in progress, whose purpose is to facilitate evaluation of the usefulness of ultrasound for LS. Further work is needed to evaluate the validity and reliability of these measures, and to identify which parameters and scoring levels are most helpful for patient care.

## Methods

### Standardization of image acquisition carried out

Two meetings were held at HUMC in 2007 (5/11/07, 7/22/07) to demonstrate image acquisition, and allow LOCUS radiologists and sonographers to review LS images, and practice image acquisition on patient volunteers. A preliminary technique protocol was developed by MSL, AM, and SCL. This protocol was reviewed and modified at the Feb 21-24, 2009 meeting, with demonstrations and supervised image acquisition sessions on patient volunteers over three days. Collected images from these sessions were jointly reviewed to enable correction of any technique errors, followed by additional demonstration and practice sessions. Dr. Liebling, Ms. Kornyat, and Mr. Mohanta were primary instructors for these sessions. During these meetings, all participants used Acuson Sequoia 512 machines, with linear-array transducers and frequencies ranging from 8 to 15 MHz, to acquire images.

### US imaging protocol

The clinicians specify which site(s) to image on the patient. Depending upon the transducer head size and lesion size, only a portion of large lesions may be able to be evaluated in a given scan. For large lesions, the clinician identifies the portion of the lesion she thinks is most active as the area to be imaged.

Ultrasound images are acquired using linear transducers, with preset software parameters for small parts, and frequencies ranging from 8 to 15 MHz. Because the frequencies used do not allow discrimination of the epidermis, we use the term dermis to refer to the combined epidermis and dermis. The operator chooses the highest frequency that allows adequate penetration; lower frequencies may be needed to image the deeper tissue layers.

Identifying sonographic changes in the involved site is accomplished by comparing both echogenicity and color Doppler signal to a corresponding normal site. For a unilateral lesion, the unaffected contralateral site is used for comparison. Both the lesion and normal control site images are viewed simultaneously on a dual image. The transducer is held perpendicular to the skin, the settings are maintained between the involved and control side, and great care is taken to match underlying landmarks. Focal zones are placed to optimize visualization of the tissue layer, and multiple zones are used for multiple layers. A thick layer of ultrasound gel is applied to improve near field visibility and avoid tissue compression, which would alter measurements of tissue thickness, echogenicity, and vascularity [25]. Gel is felt to be superior to a stand-off pad because of its ease of application and applicability on all imaging locations including the face and other non-flat surfaces. The layer of gel above the lesion and control site should be visible on the monitor.

Echogenicity differences are evaluated from grey scale images, and vascularity differences from standard color Doppler images. The sensitivity of the color Doppler is maximized by setting parameters to detect low flow. In addition, the scale is minimized until noise is reached and then raised until a visibly acceptable signal to noise ratio is reached. This typically ranges from 0.005 and 0.015. We consider each discrete color Doppler signal to represent an individual blood vessel, and refer to the color Doppler signal as indicative of vascularity in this paper. In a typical lesion scan, 9 to 20 grey scale and color Doppler dual images are acquired.

### Standardization of image interpretation

During the 2007 LOCUS meetings, radiologists, sonographers, and clinicians reviewed ultrasound images acquired from 8 LS patients followed at Hackensack University Medical Center (HUMC), comparing active to inactive patients. A preliminary ultrasound disease activity measure was generated towards the end of 2007 with the intent of capturing the range of observed differences and facilitating more consistent image interpretation. All subsequent HUMC LS patient scans collected from 1/08 to 2/09 were evaluated by MSL and SCL using this preliminary measure. Sixteen patients had ultrasound scans acquired on 51 separate dates between 1/08 to 2/09, with between one to six lesions or portions of lesions imaged per patient scan date. Approximately 90 of the scans acquired during this time were considered to be of active lesions, and 55 of inactive or minimally active lesions. Most of the scans acquired prior to this time (> 80) were also reviewed with this measure. Overall, 21 pediatric LS patients had ultrasound studies between 10/05 to 2/09, with all scans acquired on Acuson Sequoia 512 machines using linear-array transducers. Seven of the patients had circumscribed morphea, six had linear scleroderma, six had mixed morphea (both circumscribed morphea and linear scleroderma), one had generalized morphea, and one had pansclerotic morphea. From 2005 thru 2/09, the technique for acquiring images evolved to enable more consistent image quality. For our assessment of the developed measure, we only evaluated scans that were considered to have satisfactory technical quality. Similarly, only scans and images that had satisfactory technical quality were collected for group review at the February 2009 meeting.

At the February 21-24, 2009 meeting, LOCUS radiologists and sonographers reviewed over 20 LS scans plus additional dual images from multiple other scans. The meeting began with a review of the previously generated ultrasound disease activity measure, and included examples from HUMC LS patient scans of the scoring levels of each parameter of the measure. Additional patient images were then reviewed that varied between each other in observable degree of echogenicity or color Doppler differences, but would still be scored at the same level according to the developed measure. There was uniform consensus that scoring levels should be expanded to better capture the observed range of differences. The group agreed to expand color Doppler scoring to include decreased signal (-1), and broaden scoring of increased signal from 1 to 2 to 1 to 3. Scoring of echogenicity differences was expanded to include decreased echogenicity. Increased echogenicity scoring was expanded from 1 to 2 to 1 to 3 for the hypodermis layer only as the greatest range of difference was seen in this layer. There was less agreement among the group members on how to describe the different scoring levels, especially for vascularity. Several definitions were proposed, reviewed against different images, and through repeated discussions, modified until uniform consensus was achieved. MSL went through scoring of two patient scans with the group, and then each group member individually scored an additional two scans. MSL led a group review of the scoring of the second set of scans; individuals were asked how they scored the different parameters to assess the uniformity in scoring and to comment if they did not agree. The various group members reported good consistency in scoring these two scans; however these scores were not saved or formally analyzed. A reliability of scoring study was conducted during the remainder of the meeting, with each member scoring the same set of 17 scans provided to them in a random order. The results of that study will be reported at a later time. The group also defined scoring levels for evaluating tissue thickness differences and monitoring changes in lesion size. This study was approved by the HUMC institutional review board and is endorsed by CARRA.

## Results and Discussion

### Development of the Disease activity measure

Our earlier work suggested that altered echogenicity and vascularity signals in ultrasound scans of localized scleroderma patients can be associated with disease activity [9,10]. To better assess the correlation between these signals and disease activity, the LOCUS ultrasound group has been working to standardize interpretation of the observed differences. A semiquantitative scoring measure was developed following review of patient images at meetings in 2007. This measure was evaluated on subsequently collected patient scans, and modified during the February 2009 meeting. This ultrasound disease activity measure (Appendix 1) reflects the observed range of findings to date; scoring levels were based on group consensus of readily identifiable differences in sonographic echogenicity and/or vascularity. For vascularity, we score for observed differences in both number and size of discrete color Doppler signals. A greater weight is given to larger color Doppler signals as they may represent the enlarged or tortuous vessels seen in histological studies of lesions [15]. In addition, we score for relative area of color Doppler signal; this type of scoring was also used in a rheumatoid arthritis activity scoring measure [26].

Each tissue layer: dermis, hypodermis, and deep tissue (i.e., muscle, breast glandular tissue, tendon), is scored separately. The measure scores for the greatest observed difference between the lesion and normal tissue layer. The scored differences do not need to be evenly distributed in the evaluated tissue layer, and may be confined to a limited portion of the lesion. The scored echogenicity and vascularity differences must be seen in at least two dual images from a given scan to be sure that they accurately reflect the sonographic changes of the lesion. If a given difference is not seen in two images, then the individual scores of two separate dual grey scale images, or three separate dual color Doppler images, are averaged for scoring echogenicity or vascularity, respectively. The cumulative disease activity score is determined by summing the absolute value of the individual parameters. This yields a range from 0 to 15 (maximum echogenicity score of 6, maximum vascularity score = 9, Appendix 1). Further study is needed to determine if a -1 score for echogenicity and/or vascularity has different significance for disease activity than a +1 signal. It may be that a -1 vascularity score, for example, represents loss of normal vasculature secondary to fibrosis and is therefore more reflective of late stage changes or damage than activity.

**Figure 1:**
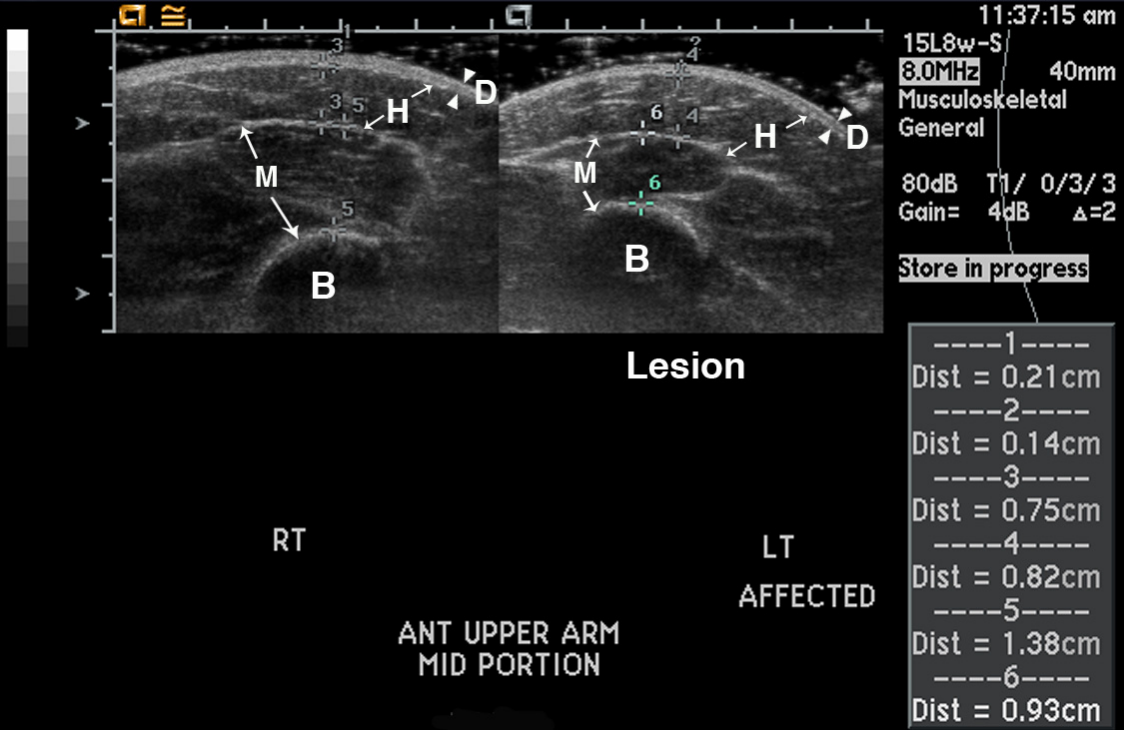
**14 year old girl with linear scleroderma affecting her left arm; portion shown is from her upper arm**. She has chronic atrophy from long standing disease (11 years), with persistent signs of disease activity including erythema and pruritis. Ultrasound shows varying patterns of echogenicity differences in the lesion, with no difference found in the dermis (dermis echogenicity = 0), mildly increased echogenicity in the hypodermis in a patchy pattern (hypodermis echogenicity = 1), and decreased echogenicity in the muscle (deep tissue echogenicity = -1). Ultrasound allows facile measurement of tissue thickness. Cursors were placed at tissue boundaries, using the highest point of the humeral bone as a landmark for the measurements. The lesion dermis is thinner than the normal dermis (measurement 2 (0.14 cm) vs 1 (0.21 cm), respectively; dermis tissue thickness score 1). The lesion hypodermis is mildly thicker than the normal hypodermis (measurement 4 (0.82 cm) vs 3 (0.75 cm), respectively; hypodermis tissue thickness score -1). The lesion muscle is thinner than the normal muscle (measurement 6 (0.93 cm) vs 5 (1.38 cm), respectively; deep tissue tissue thickness score 2). B = bone, D = dermis, H = hypodermis, and M = muscle. Arrowheads indicate boundaries of dermis, while arrows indicate boundaries of hypodermis and muscle. Large tick marks on x and y axis = 1 cm.

**Figure 2:**
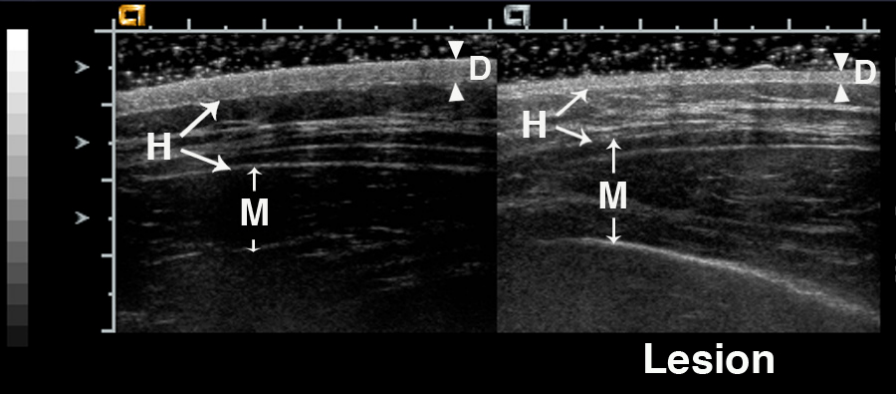
**17 year old girl with generalized morphea that began over 13 years earlier**. She has lesions on her back, abdomen, arms, and leg. Most lesions show dyspigmentation and mild tissue loss, but her back lesions have increased warmth. Ultrasound of a back lesion shows thinning of the dermis and hypodermis, and an increase in lesion dermal echogenicity (dermis echogenicity = 1). There are highly echogenic horizontal bands in the hypodermis layer of both the normal and lesion hypodermis layers. The echogenicity of these bands is increased in the lesion, as is the base level of hypodermal echogenicity (hypodermis echogenicity = 2). The lesion muscle shows a patchy increase in echogenicity compared to the normal muscle (deep tissue echogenicity = 1). Arrowheads indicate boundaries of dermis, while arrows indicate boundaries of hypodermis and muscle.

**Figure 3:**
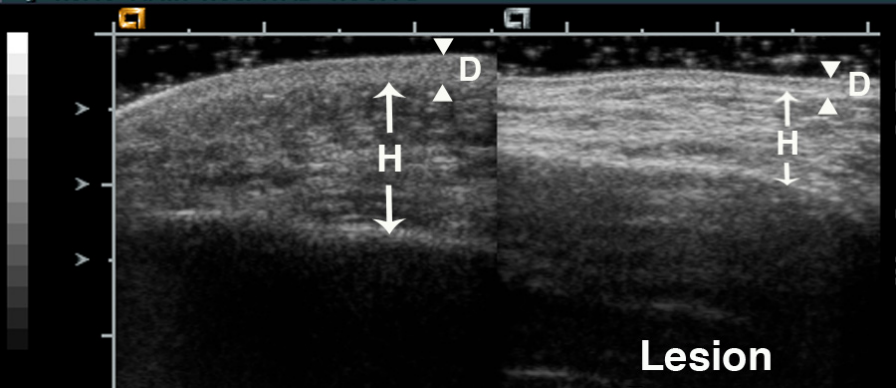
**14 year old boy with circumscribed deep morphea of the infraorbital region for 2 years**. His lesion has shown deeper pigmentation changes and recent increased tissue loss. There is marked thinning of the hypodermis layer. The echogenicity of the hypodermis is markedly increased, and greater than the echogenicity of the normal dermis (hypodermis echogenicity = 3). The echogenicity of the lesion dermis is also increased (dermis echogenicity = 1). D = dermis, H = hypodermis. Arrowheads indicate boundaries of dermis, while arrows indicate boundaries of hypodermis.

The largest range of echogenicity differences was seen in the hypodermis and smallest in the dermis; echogenicity differences are scored on a -1 to 1 range for dermis, -1 to 3 range for hypodermis, and -1 to 2 range for deep tissue (Appendix 1). A score of -1 represents hypoechogenicity, 0 isoechogenicity, and positive numbers hyperechogenicity of the lesion relative to the normal tissue layer. A hypodermis echogenicity score of 3 was defined as echogenicity of the hypodermis equal to or greater than that of the normal dermis. To aid scoring of lower levels of echogenicity, the scorer can compare with visual examples such as those in Figures 1 to 3.

Figures 1, 2, 3 show examples of different echogenicity scoring levels. The lesion dermal echogenicity is increased compared to normal dermis in Figures 2 and 3 (dermal echogenicity score of 1). Figure 1 shows a mild increase in hypodermal echogenicity (score of 1) in a patchy distribution, while Figures 2 and 3 show a more uniform increase in hypodermal echogenicity. A moderate increase in hypodermal echogenicity is seen in Figure 2 (score of 2), and a marked increase in Figure 3 (score of 3). Figure 1 shows a decrease in lesion muscle (deep tissue) echogenicity compared to the normal muscle (score of -1), while Figure 2 shows a mild increase in muscle echogenicity (score of 1) in a patchy pattern.

Vascularity differences are scored on a -1 to 3 range for all tissues, where 0 represents no difference in color Doppler signal level between the lesion and normal site, and is defined as the range in color Doppler signal between -1 and 1. A score of -1 represents at least two fold fewer color Doppler signals in the lesion compared to the normal site, 1 represents up to two fold more color Doppler signals or one more large color Doppler signal in the lesion than normal site, and 2 and 3 represent further increases in the lesion color Doppler signal level compared to the normal site (Appendix 1). The maximum score of 3 requires at least four more large color Doppler signals, or an area of color Doppler signal involving at least 20% more of the lesion tissue layer, compared to the normal layer.

**Figure 4:**
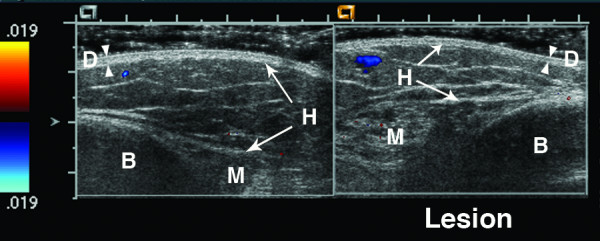
**15 year old girl with linear scleroderma of her left leg that has progressed over the past three years**. Her calf lesion shows erythema, warmth, and continuing tissue loss. Ultrasound shows an increase in lesion hypodermal vascularity; there are a few small color Doppler signals in both the lesion and normal hypodermis, and one large color Doppler signal in the lesion hypodermis (hypodermal vascularity = 1). D = dermis, H = hypodermis. Arrowheads indicate boundaries of dermis, while arrows indicate boundaries of hypodermis.

**Figure 5:**
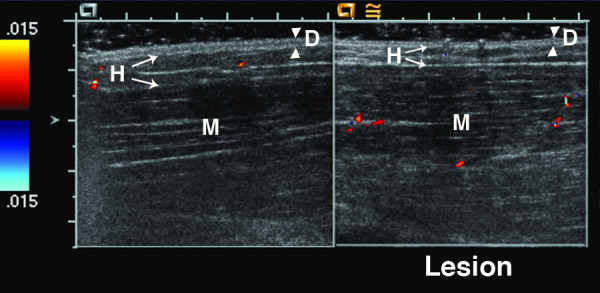
**15 year old girl with over an 8-year history of localized scleroderma**. She initially presented with linear scleroderma of one hand that extended up her arm. There has been recent extension of this lesion to her upper back, and circumscribed morphea lesions have developed on her chest and thigh. Ultrasound image shown is of her upper arm, which had chronic atrophy and hyperpigmentation, but also mild erythema. There is increased vascularity in the lesion muscle, with over 10 discrete color Doppler signals in this layer versus none in the normal muscle. The deep tissue vascularity is scored as 2 because there are over two-fold more color Doppler signals in the lesion compared to normal; the vascularity is not scored as 3 because all of the lesion color Doppler signals are of a similar small size. The normal hypodermis has over two-fold more color Doppler signals than the lesion in this image, giving a lesion hypodermal vascularity score of -1. D = dermis, H = hypodermis, and M = muscle. Arrowheads indicate boundaries of dermis, while arrows indicate boundaries of hypodermis.

**Figure 6:**
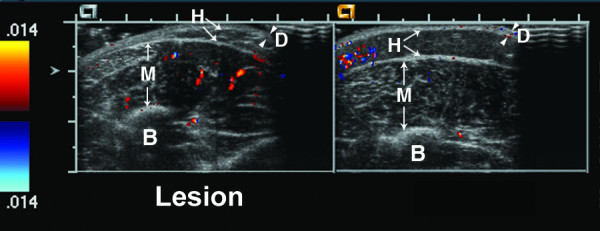
**6 year old girl with linear scleroderma of her calf for the past two years**. The lesion is warm and mildly violaceous. There is loss of hypodermal fat in the lesion (left side of lesion image). The lesion muscle layer has increased vascularity with at least 4 more large color Doppler signals on the lesion side compared to the normal muscle (deep tissue vascularity = 3). The Doppler signal seen on the left hand side in the normal hypodermis layer is artifact. B = bone, D = dermis, H = hypodermis, and M = muscle. Arrowheads indicate boundaries of dermis, while arrows indicate boundaries of hypodermis and muscle.

**Figure 7:**
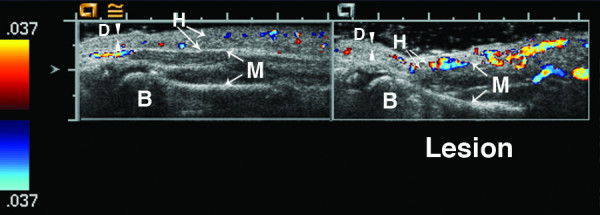
**6 year old girl with a four year history of linear scleroderma of her left arm and hand**. Her lesion has extended to her upper back, and she has new lesions on her face and chest. The imaged area is the volar surface of hand above 5^th ^metacarpal bone, which shows chronic atrophy including shortening of this portion of her hand, but no signs of activity. The patient complained of pain in this area. There is abundant color Doppler signal in both the normal and lesion area, but more vascularity is seen in the lesion hypodermis and muscle layers. The lesion hypodermis shows a large area of contiguous color Doppler signal that encompasses at least 20% of the surface area of the imaged hypodermis (hypodermal vascularity = 3). There are 2 large color Doppler signals in the lesion muscle and none in the normal muscle, giving a deep tissue vascularity score of 2. B = bone, D = dermis, H = hypodermis, and M = muscle. Arrowheads indicate boundaries of tissue layers.

Figures 4, 5, 6, 7 show examples of different vascularity scoring levels. In Figure 4, there are similar numbers of small color Doppler signals between the lesion and normal hypodermis, and one large color Doppler signal in the lesion giving a hypodermal vascularity score of 1. In Figure 5, there are fewer color Doppler signals in the lesion compared to normal hypodermis (hypodermal vascularity score of -1), but more color Doppler signals in the lesion muscle (deep tissue vascularity score of 2). The same maximum vascularity score of 3 is given to lesion tissue layers in Figures 6 and 7, with this score achieved by number of large color Doppler signals in Figure 6, and by surface area of color Doppler signal in Figure 7.

### Tissue thickness and lesion size assessments

Although several studies have reported that changes in dermal thickness are associated with changes in disease state (worse vs improved [6-8]), we have not found an obvious association between dermal, hypodermal, or deep tissue thickness changes and disease activity in our patients. In both active and inactive lesions, the lesion tissue layer was usually thinner than or comparable in thickness to the normal layer. Tissue thickening was seen in some lesions, which might represent edema for newer lesions and fibrosis for older lesions. We have, therefore, decided to evaluate tissue thickness changes separately from echogenicity and vascularity changes to better determine the relationship between thickness changes of each tissue layer and disease activity (Appendix 2). It may also be that monitoring changes in tissue thickness over time, rather than simply the presence of tissue thickening or thinning, will aid disease activity assessment.

Tissue thickness differences between the lesion and normal site are scored separately for each observable tissue layer, with scoring including both thickening (-1) and thinning (ranging from 1 to 3). The scoring range is greatest for the hypodermis because the largest variation was seen in this layer, ranging from mild to complete loss of the hypodermal fat (Figure 1). Further study is needed to learn if thickening and thinning are equivalent in order to determine if the individual tissue layer thickness scores, or their absolute values, should be added together. Measurements are obtained at the greatest difference in thickness compared to the normal site. Great care is taken to insure the underlying landmarks such as muscle planes and bone are comparable from side to side (Figure 1). If no sonographically distinguishable landmark is present, external landmarks, such as distance from a joint are used for determination of transducer placement. Measurements are obtained either at the time of imaging acquisition or when viewing subsequently (Figure 1).

**Figure 8:**
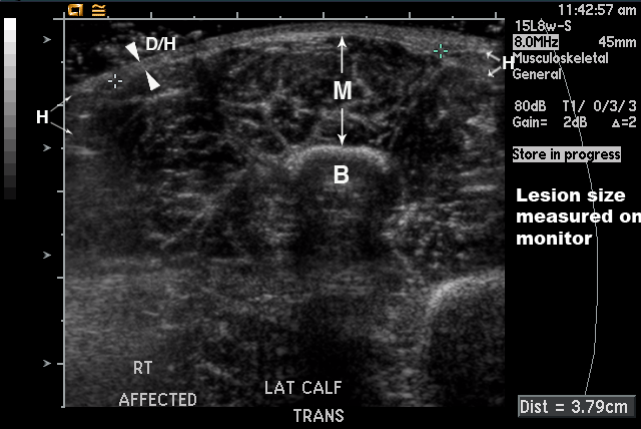
**8 year old girl with linear scleroderma of her lower leg with a four year history of active disease**. A single rather than dual screen image was obtained to allow measurement of lesion size. The boundaries of the area of complete loss of hypodermal fat were identified directly on the viewing monitor, with cursors placed at boundary points to determine the width of the area of fat loss (3.79 cm). B = bone, D = dermis, H = hypodermis, and M = muscle. Arrowheads indicate boundaries of merged dermis and hypodermis, while arrows indicate boundaries of hypodermis and muscle.

We will also evaluate interval changes in overall lesion size in comparison to disease activity. Here, we define lesion size as the area of complete hypodermal fat loss. This lesion size score is, therefore, only applicable in a subset of patients, namely those who have a discretely measurable area of hypodermal fat loss. The lesion boundaries must be accessible to ultrasound, so the size of lesions involving the scalp, near the eye or nose, or similar locations is not obtainable. The maximum length and maximum orthogonal width are measured; if the lesion size is smaller than the size of the transducer head, the length and width can be directly measured on the viewing monitor. In this case, single rather than dual images can be acquired as shown in Figure 8. If the lesion is greater than the transducer head, then the boundaries of complete hypodermal loss are identified sonographically, marked on the patient with ink, and size measured with a tape measure. Both worsening (increase in one or both dimensions) and improvement (decrease in one or both dimensions) can be documented with this score.

## Conclusion

Localized scleroderma remains a challenging disease to treat, with the potential for severe morbidity and disability. There is no consensus on treatment, which ranges from topical agents to phototherapy to systemic immunosuppressive medications (reviewed in [4,27]). A great need exists for sensitive disease assessment tools to better evaluate treatment efficacy.

We and others have found that ultrasound can detect several differences between active localized scleroderma lesions and normal tissue. We have observed a range of differences in tissue echogenicity between the lesion and normal side, from hypoechoechogenicity to isoechogenicity to hyperechogenicity, with active lesions more likely to show hypo- or hyperechogenicity than inactive lesions. Similarly, we have observed a range of differences in tissue vascularity between the lesion and normal side, from decreased vascularity to no difference to marked hyperemia. The ultrasound frequencies we use allow detection of differences in the dermis, hypodermis, and deep tissue layer, but not in the epidermis. For children, evaluation of the hypodermis and deep tissue layers may be especially important as disease often involves the deeper tissues, putting the child at risk for major growth disturbances.

As an initial step towards developing ultrasound as an assessment tool for localized scleroderma, our group has been working to standardize image acquisition and interpretation for these patients. Training and practice imaging sessions on patient volunteers were held to develop a standard image acquisition protocol that we present here. Joint reviews of patient images were held at meetings in 2007 and 2009, and led to consensus on scoring measures to facilitate more consistent interpretation of alterations in lesion echogenicity, vascularity, and tissue thickness. A limitation of our study is that we did not have each radiologist and sonographer independently review images, but only had a group review prior to discussions leading to consensus. We also did not consider the radiologists' and sonographers' prior experience in evaluating ultrasound images when working towards consensus. Our aim, however, was to standardize ultrasound acquisition and image assessment within our group so that we could collectively work to evaluate ultrasound assessment of localized scleroderma. These measures should be considered preliminary. Scoring levels were set based upon the observed range of sonographic differences, and further study is needed to evaluate their validity and reliability and determine clinically relevant scoring levels and changes. Once valid and reliable tools are developed, clinical trials can be conducted to work towards improved treatment and outcome for these patients.

## List of Abbreviations

CARRA: Childhood Arthritis and Rheumatology Research Alliance; HUMC: Hackensack University Medical Center; LOCUS: Localized scleroderma clinical and ultrasound study group; LS: localized scleroderma.

## Competing interests

The authors declare that they have no competing interests.

## Authors' contributions

SCL and MSL organized the meetings, and reviewed all of the patient scans. MSL acquired all the scans with assistance of TK. FR, MSL, SCL, AD, AM, and SZ developed the initial version of the measures. All of the authors participated in discussions that led to the currently proposed versions. MSL, AM, and SCL developed the initial acquisition protocol, with all authors reviewing and approving the final protocol. SCL and MSL wrote the manuscript, which has been approved by all the authors.

## Appendix 1: Ultrasound disease activity measure (U-DA)

### Echogenicity Score

Dermis:

-1 = Decreased compared to normal site

0 = No difference compared to normal site

+1 = Increased compared to normal site

Hypodermis:

-1 = Decreased compared to normal site

0 = No difference compared to normal site

+1 = Some increase compared to normal site

2 = Moderate increase: echogencity > normal hypodermis but < normal dermis signal

3 = Large increase: echogenicity similar to or > normal dermis signal

Deep tissue (i.e., muscle, tendon, glandular)

-1 = Decreased compared to normal site

0 = No difference compared to normal site

+1 = Mild increase compared to normal site

2 = Moderate to large increase compared to normal site

### Vascularity Score

0 = No difference compared to normal site (between 1 and decreased)

1 = Mild; lesion has 1 more larger vessel than normal site

Or, if no or same number of larger vessels present, then lesion has more

blood vessels then normal site (ratio of lesion to normal blood vessels >1x, ≤ 2x)

2 = Moderate; in between 1 and 3; lesion has 2 or 3 more larger vessels

compared to normal site. Or, if no or same number of larger vessels

present, then lesion has >2x more blood vessels than normal site

3 = Large; lesion has ≥ 4 larger vessels than normal site

Or, lesion has increased number and/or size of blood vessels with increase involving at least 20% more of tissue layer field area

-1 = opposite of 2; Normal site has ≥ 2 larger vessels or >2x more blood vessels than lesion

Score each visualized layer separately. Not all lesions have a deep tissue layer. Each parameter is scored for the greatest level of difference that is seen in at least two separate dual images. The scored differences do not need to be evenly distributed in the evaluated tissue layer. If a given difference is not seen in two images, then the individual scores of two separate dual grey scale images, or three separate dual color Doppler images, are averaged for scoring echogenicity or vascularity, respectively. Each color Doppler signal is considered to represent a blood vessel. Dermis represents both epidermis and dermis. When there is complete loss of subcutaneous fat within the hypodermis, the dermis and hypodermis are not visually separable; the layer superficial to a region of complete hypodermal fat loss is scored as dermis. In this case, hypodermal echogenicity is scored from hypodermis directly adjacent to the site of complete loss. The absolute values of the individual parameters are summed to determine the score, which ranges from 0 to 15.

## Appendix 2: Tissue thickness and Lesion size scores

### Tissue Thickness Score

Dermis

-1 = thickening

0 = none

1 = thinning

Hypodermis

-1 = thickening

0 = none

1 = mild thinning, <20%

2 = thinning ≥ 20% to 89%

3 = ≥ 90% thinning, or no fat

Deep Tissue

-1 = thickening

0 = none

1 = thinning <20%

2 = thinning ≥ 20%

### Lesion Size Score

percentage change refers to a change in measured length or width

-2 = decreased >25%

-1 = decreased 10-25%

0 = <10% change in size

+1 = increased 10-25%

+2 = increased >25%

The tissue thickness score is determined by evaluating the degree of tissue loss or thickening in the lesion dermis, hypodermis, and, if present, deep tissue layer, in comparison to the normal tissue layers. Measurements are obtained at the most abnormal thickness portion of the lesion using landmarks such as a bone to aid identification of the normal comparison site. Measurements can be obtained at the time of imaging acquisition or during subsequent review.

The lesion size score can only be used on lesions that have a sonographically accessible area of complete loss of subcutaneous fat. The maximum length and maximum orthogonal width are measured; if the lesion size is smaller than the size of the transducer head, the length and width can be directly measured on the viewing monitor. Otherwise, boundaries are identified by ultrasound, marked on the patient's skin, and then measured with a tape measure. Negative numbers indicate a decrease in lesion width and/or length as would be seen with clinical improvement, and positive numbers indicate an increase in width and/or length as would be seen with disease flare or worsening.
